# Accelerometer-based physical activity levels, fundamental movement skills and weight status in British preschool children from a deprived area

**DOI:** 10.1007/s00431-019-03390-z

**Published:** 2019-05-07

**Authors:** Clare M. P. Roscoe, Rob S. James, Michael J. Duncan

**Affiliations:** 1 Human Sciences Research Centre, University of Derby, Kedleston Road, Derby, DE22 1GB UK; 2 Centre for Applied Biological and Exercise Sciences, Coventry University, Priory Street, Coventry, CV1 5FB UK

**Keywords:** Physical activity, Fundamental movement skills, Preschool children, GENEActiv Accelerometer, Test of Gross Motor Development-2

## Abstract

Preschool children are recommended to participate in a minimum of 180-min physical activity (PA) per day to enhance their development and overall health. Low PA and increased obesity are thought to be linked to low mastery of fundamental movement skills (FMS) in preschool children. This study sought to investigate whether FMS influences PA levels and weight status in preschool children, in an area of low socioeconomic status. Secondary aims of this study were to determine whether gender or day of the week affected the primary outcomes. One hundred eighty-five preschool children aged 3–4 years old, participated in the study. FMS proficiency was determined using the Test of Gross Motor Development-2. PA was determined using triaxial accelerometry over a 4-day period. None of the samples met the recommended 180 min of PA. There were no significant differences in PA or weight status between preschool children with high, medium or low FMS mastery (*P* < 0.05). There were also no significant correlations between overall FMS and moderate to vigorous PA during the week or weekend days.

*Conclusion*: Girls scored significantly greater at the hop, leap, and skip (locomotor skills) and the boys significantly higher at the kick (object control) (*P* < 0.05). There were no significant differences in PA or weight status between preschool children with high, medium, or low FMS mastery, possibly because FMS mastery had not developed to a high enough level to affect PA and FMS are considered independent of physical fitness and physical features, such as weight and height.

**Table:** 

**What is Known**: •*FMS are commonly developed in early childhood, providing the building blocks for future motor skills, good health and lifelong PA*. •*No study to date has measured FMS, PA levels and weight status in preschool children, to determine whether FMS competency influences PA levels and weight status in preschool children, in an area of low SES*. **What is New**: •*FMS competency did not appear to influence the level of PA or weight status in this sample of UK preschool children from a low SES area*. •*PA and FMS may not be fully established and consequently not strongly linked at the ages of 3–4 years, therefore, the preschool years could be influential in providing a window to maximise input of good/optimal development of motor competence before the proficiency barrier sets in and we need remedial intervention*.

**What is Known**:

•*FMS are commonly developed in early childhood, providing the building blocks for future motor skills, good health and lifelong PA*.

•*No study to date has measured FMS, PA levels and weight status in preschool children, to determine whether FMS competency influences PA levels and weight status in preschool children, in an area of low SES*.

**What is New**:

•*FMS competency did not appear to influence the level of PA or weight status in this sample of UK preschool children from a low SES area*.

•*PA and FMS may not be fully established and consequently not strongly linked at the ages of 3–4 years, therefore, the preschool years could be influential in providing a window to maximise input of good/optimal development of motor competence before the proficiency barrier sets in and we need remedial intervention*.

## Introduction

Fundamental movement skills (FMS) are commonly developed in early childhood, providing the building blocks for future motor skills, good health and lifelong physical activity (PA) [32, 56]. Impaired FMS development, in early childhood, has been reported to be associated with lower PA and increased obesity [9]. These lower PA levels are highlighted by only 9% of preschool boys and 10% of preschool girls in England, meeting the Chief Medical Officer’s recommendations for 180 min of PA per day [10, 26]. However, no significant relationship has been identified between FMS and body mass index (BMI) in preschool children [31, 34, 36], suggesting that both obese and overweight preschool children develop FMS competency at the same rate as healthy weight preschool children. However, the relationship between FMS and BMI remains equivocal [34] and further research is required to determine the exact link between these.

Stodden et al.’s (2008) [51] conceptual model suggests that as children age, those with intermediate to higher levels of motor competence and greater PA levels will demonstrate higher performance scores in terms of both locomotor and object control skills [51], compared to those with less developed FMS. Literature supports the conceptual model [51], in that during the developmental stage (preschool years), the association between motor competency and PA is weak, but that developing FMS competency is important in reducing sedentary behaviour and increasing PA [22]. Preschool children with better-developed motor skills spend significantly more time in moderate to vigorous PA (MVPA) and significantly less time in sedentary behaviours, than children with less developed motor skills [59]. Improving FMS competency will allow preschool children to complete higher levels of PA [27] and this should aid in reducing the risk and rate of being overweight or obese and its associated diseases [43]. This is an area which requires further research to identify if differences in FMS competency influence PA levels.

A child of low socio-economic status (SES) has a greater chance of a delay in their FMS development [24], suggesting they are at a greater risk of being obese. Young children from deprived areas are considered to have limited access to safe outdoor play areas or lack opportunities to engage in activities which help to promote and foster FMS [19, 20] and have lower PA levels [50]. Additionally, preschool children are reported to display different activity patterns on weekdays, compared to weekend days, with both genders being more physically active on the weekdays [6] and boys engaging in MVPA significantly (*P* < 0.001) more than girls [27]. This study aims to measure FMS, PA levels and weight status in preschool children, to determine whether variation in FMS competency is related to variation in PA levels or weight status in preschool children, in an area of low SES. Secondary aims of this study are to determine whether gender or day of the week influenced the primary outcomes, as has been found in older children [46].

## Method

### Participants and data collection

Following institutional ethics approval from Coventry University (P45654) and informed consent, children from 11 preschools in North Warwickshire, England participated in this study; of these preschools, five were private and six were on a primary school site. Children’s assent was gained through the desire to be involved in the testing and those unwilling to participate were removed from the study. The participants were a convenience sample and included 185 preschool children (99 boys, 86 girls), aged 3–4 years. North Warwickshire was chosen as it incorporates preschools that are considered to have the highest levels of deprivation in the County [58]. The preschools selected were all in an area of SES deprivation.

### Anthropometric assessment

Height was measured to the nearest millimetre, using a portable stadiometer (Leicester Height Measure, Leicester, UK). Body mass was measured to the nearest 0.1 kg using portable weighing scales (Tanita BF350, Tokyo, Japan); the children were lightly dressed (t-shirt and light trousers/skirt) and barefoot, or in socks. BMI was calculated as kilograms per square meter [53]. BMI was compared to a BMI-for-age chart to determine whether the child was of normal weight or overweight (≥ 95th percentile); this is recommended as a reasonable measure for assessing overweight in children [2, 4, 11, 28]. Waist circumference (WC) was measured to the nearest centimetre, midway between the lowest rib and the iliac crest [30], using a non-elastic flexible tape measure and the child in a standing position. WC was compared to standardised international cut-off points and weight status categorised as overweight/obese or normal weight [33].

### Assessment of physical activity

PA was measured using a GENEActiv waveform triaxial accelerometer (ActivInsights Ltd., Cambridge, UK). The accelerometer measured at 10 epochs (s) and a sampling frequency of 100 Hz [25, 41, 55]. The accelerometer was attached using a watch strap and positioned over the dorsal aspect of the dominant wrist, midway between the radial and ulnar styloid process. The participants wore the accelerometers for four consecutive days; this included 2 weeks and two weekend days [52]. All children received a letter to take home describing how and when they should wear the GENEActiv accelerometers. Non-wear time was defined as 90-min windows of consecutive zero or nonzero counts [7]. The amount of wear time and percentage (%) of wear time that each child spent in different intensities of PA were calculated for weekdays and weekend days. It is recommended that 4 days, including one weekend day, is acceptable for measuring PA [52]. Due to the age of the participants and the difficulty in children wearing accelerometers for sustained periods of time, children were included in the final data analysis if the accelerometer had been worn for 3 days, including one weekend day and for a minimum of 6 h each day [5, 47, 52]. Of the 185 children sample, accelerometer data was recorded for 178 children; seven children’s data were not useable due to the children either not wearing the accelerometers or technical difficulties with them recording data.

For every epoch (number of seconds), movement data (activity counts) were added, logged, processed and analysed. Accumulated activity counts were categorised in terms of intensity: sedentary, light, moderate and vigorous PA [1]. The following cut points for 3–4-year-olds were used to determine PA intensity: dominant hand < 8.1 cpm for sedentary activity, 8.1–9.3 cpm for LPA and 9.3+ cpm for MVPA [47]^.^ For the non-dominant hand, < 5.3 cpm for sedentary activity, 5.3–8.6 cpm for LPA and 8.6+ cpm for MVPA [47]. Using the GENEActiv post-processing software, the raw 100 Hz triaxial GENEActiv data were summed into a signal vector magnitude and expressed in 10-s epochs [18]. Children were classified as either meeting (sufficiently active) or not meeting (insufficiently active) the UK recommended 180 min a day of PA for 0–5-year-olds [10].

### Assessment of FMS

An adapted version of the Test of Gross Motor Development-2 (TGMD-2) was employed as a measure of FMS [54], with the removal of the underhand roll and the addition of skipping. Skipping was included because it is a skill that benefits children’s physical fitness, improving their balance and muscle coordination, whilst being an enjoyable low-cost activity [16, 48]. TGMD-2 is a process-orientated test that examines a subset of locomotor and object control skills [3, 23]. The TGMD-2 relates to activities that preschool children participate in and it assesses gross motor development amongst 3- to 11-year-olds [23]. The TGMD-2 has been described as having an established validity and reliability amongst preschool children, with a test-retest reliability of 0.82–0.95 [23, 38].

This study adhered to the TGMD-2 guidelines with the addition of skipping and the removal of the underhand roll [54]. Prior to data collection, a senior member of the research team, who had previous experience of delivering the TGMD-2 protocol, trained the field tester (primary researcher). The children were assessed in small groups (2–3) and the tests were administered by one tester to ensure consistency. The tests took part in an outside area or an adjacent primary school’s hall. The skills were physically demonstrated and verbally explained to ensure all children had the same clear information on the different skills. If any child did not understand a task correctly then they were provided with a further verbal description and asked to repeat the trial of the skill again [14]. The children all had a practice attempt prior to being scored on their two tests. All children were videoed completing the skills, using a camcorder (Sony, Tokyo, Japan) at standard frame rate, allowing the skills to be analysed after the occasion. All 12 skills were assessed in a standardised order and the testing took between 30 and 35 min per group. The skills were always performed in the following order: run, gallop, hop, leap, horizontal jump, skip, slide (locomotor skills), followed by two-handed strike of a stationary ball, stationary bounce of a ball, catch, kick and overhand throw (object control skills) [3, 23].

The children’s FMS competency was assessed using the guidelines of the TGMD-2 protocol [54]. All video analyses were completed by the field tester (primary researcher). Inter-tester reliability was established prior to the commencement of testing, using pre-coded videos of ten children, there was 84.5% agreement across the 12 skills (range = 81.7–88.4%); this was similar to work by Foulkes et al. (2015) [14]. Intra-tester reliability was also performed again using pre-coded videos of an additional ten children, with the test-retest completed 1 week apart, 93.9% agreement was determined across the 12 skills (range = 90–98%). There is no specified minimum level of percentage agreement, however, 80–85% has previously been deemed acceptable [57]. If at any point the assessor was unsure whether a child had performed a criterion or not, then both the assessor and trainer viewed the videos and agreed on a score [14]. For both trials of the skill, the run, gallop, hop, jump, slide, strike, catch, kick and throw were all scored out of 4 and the leap, skip and bounce were out of 3; zero represented absence of components for a skill. The scores were totalled over two attempts to provide the locomotor, object control and total gross motor skill score for a child (total FMS) [3, 23].

### Statistical analysis

The percentage of time and mean amount of time (min) spent in sedentary, LPA and MVPA were determined. Data for total, locomotive and object control FMS were analysed separately. The participants were split equally into tertiles for their total FMS, locomotive FMS and object control FMS. This was to identify whether being in the lower, middle or higher FMS tertile for these measurements impacted on PA, BMI and WC. The use of tertiles in this manner has been recommended [12, 59] in the context of motor competence research with children as the most effective way to understand whether health-related variables differ as a consequence of FMS being low, moderate or high in nature. The effect of any differences in the FMS measurements on PA, BMI and WC was analysed using a univariate ANOVA, with WC, BMI or PA as the dependent variable and gender and the specific FMS tertile as the fixed factors. The Bonferroni post hoc test was used to analyse any differences. Independent two tailed *t* tests were also performed to identify if there were any significant differences between each FMS between boys and girls. Pearson’s correlation coefficients were performed between the overall total FMS scores, locomotor FMS and object control FMS and the percentage of time in MVPA during the weekdays and the weekend days, to see if there was a relationship. Pearson’s correlation coefficient was calculated for each combination of BMI or WC against total, locomotor or object control FMS. Pearson’s correlation requires parametric data; therefore, arcsine transformation was conducted on the percentage time data (non-parametric) prior to statistical analysis [60]. The Statistical Package for Social Sciences (Version 20, SPSS Inc., Chicago, Ill, USA) was used for statistical analysis and the alpha level was set a priori at *P* = 0.05.

## Results

**Fig. 1:**
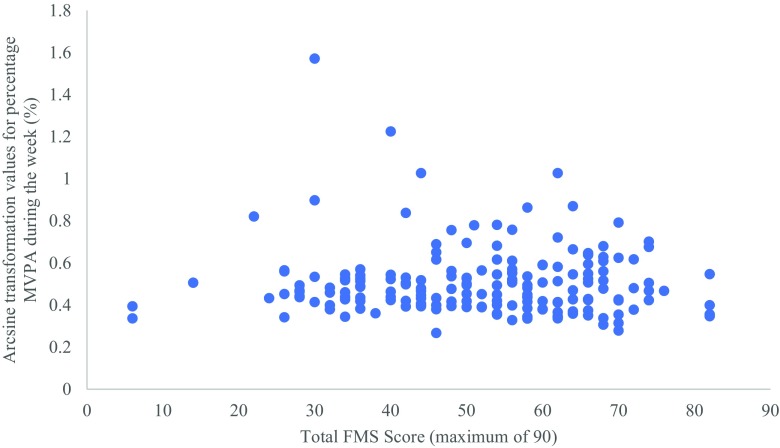
Total FMS score against the arcsine transformed values of mean percentage MVPA during the weekdays

**Fig. 2:**
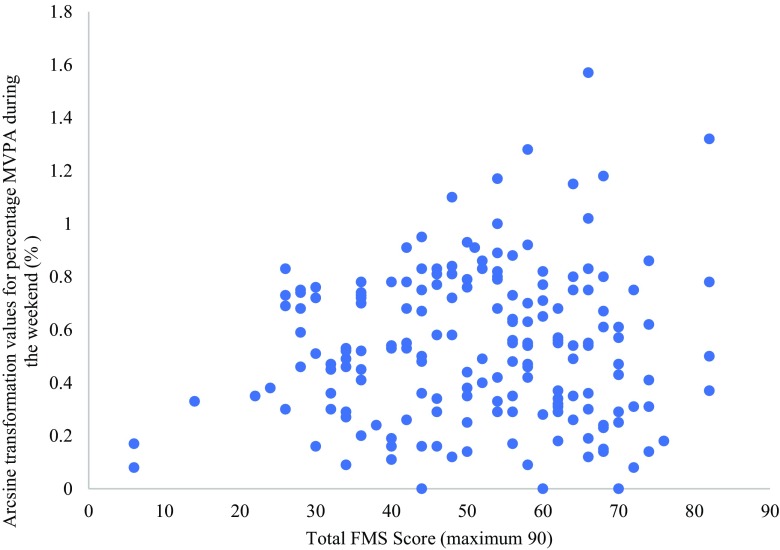
Total FMS score against the arcsine transformed values of mean percentage MVPA during the weekend days

There was no significant correlation between overall FMS and MVPA during weekdays or weekends (*r* = − .030, *P* > 0.05 for weekdays; *r* = .070, *P* > 0.05 for weekends) (Figs. 1 and 2). Equally, there was no significant correlation between locomotor FMS and MVPA during weekdays or weekends (*r* = − .002, *P* > 0.05 for weekdays; *r* = .079, *P* > 0.05 for weekends), nor between object control FMS and MVPA during weekdays or weekends (*r* = − .072, *P* > 0.05 for weekdays; *r* = .040, *P* > 0.05 for weekends). Furthermore, there was no significant correlation between BMI and total FMS, locomotor FMS and object control FMS (*r* = .038, *P* > 0.05 for total FMS; *r* = .039, *P* > 0.05 for locomotor FMS; *r* = .029, *P* > 0.05 for object control FMS), nor between WC and total FMS, locomotor FMS and object control FMS (*r* = .068, *P* > 0.05 for total FMS; *r* = .079, *P* > 0.05 for locomotor FMS; *r* = .034, *P* > 0.05 for object control FMS).

Descriptive characteristics, including overall mean time (min) spent in the different intensities of PA, are summarised in Table 1. Overall FMS and PA level for each tertile of FMS are in Table 2. Of the sample, 9.25% were classed as overweight/obese and none of the children met the UK recommended 180 min or more of PA (moderate and vigorous intensity) per day. Two, circa 1%, of the children did meet the 180 min on one of their days, but not for all days. There were no significant differences in PA levels, BMI or WC between tertiles of total, locomotive or object control FMS (all ANOVA *P* > 0.05) (Table 2).

**Table 1:** Children’s descriptive characteristics. Data represent mean ± SD, *n* = 178

Age (years)		3.4 ± 0.5
Mass (kg)		16.8 ± 2.5
Height (cm)		101.7 ± 4.8
Body mass index (kg m^2^)		16.3 ± 1.9
Waist circumference (cm)		55.0 ± 3.9
Normal body mass index		90.75%
Overweight/obese body mass index		9.25%
Mean wear time (min) during the week and weekend		577.0 ± 90.0
Mean sedentary behaviour (min) during the week and weekend		545.0 ± 85.0
Mean light PA (min) during the week and weekend		9.0 ± 12.0
Mean moderate and vigorous PA (min) during the week and weekend		25.0 ± 14.0
Sedentary behaviour (%) during the week and weekend		94.2 ± 2.8
Light PA (%) during the week and weekend		1.5 ± 1.8
MVPA (%) during the week and weekend		4.3 ± 2.3
Met PA guidelines of at least 180 min per day total PA (%)	Sufficiently active	0
	Insufficiently active	100

**Table 2:** Overall range for total FMS (0–90), percentage of time spent in MVPA, BMI and WC for each tertile. Data represent mean ± SD. *n* = 178

	FMS tertile		
	Low tertile	Medium tertile	High tertile
Overall FMS	34.0 ± 8.5	52.7 ± 4.3	67.5 ± 5.7
PA levels (percentage of time in MVPA weekdays)	1.87 ± 0.17	2.31 ± 0.14	3.10 ± 0.58
PA levels (percentage of time in MVPA weekend days)	0.23 ± 0.10	0.52 ± 0.08	0.85 ± 0.17
BMI (kg/m^2^)	14.2 ± 1.5	16.4 ± 0.3	18.2 ± 1.2
WC (cm)	51 ± 1.3	54 ± 0.9	59 ± 3.1
Mean Age (years)	3.35 ± 0.48	3.37 ± 0.48	3.38 ± 0.49

Girls scored significantly greater at the hop, leap and skip (locomotor skills) and the boys significantly higher at the kick (object control) (*P* < 0.05; Table 3). The girls tended to score higher on total locomotor FMS (Table 4; *P* = 0.08) and the boys were significantly higher on total object control FMS (*P* < 0.05). There was no significant difference in total FMS score between boys and girls (Table 4).

**Table 3:** FMS competency score of the 12 FMS. Data represent mean ± SD

	Locomotive FMS (0–52 is possible)			Object Control FMS (0–38 possible)		
	All participants (*n* = 178)	Boys (*n* = 97)	Girls (*n* = 81)	All participants (*n* = 178)	Boys (*n* = 97)	Girls (*n* = 81)
Run	7.3 ± 1.2	7.2 ± 1.4	7.4 ± 1.1			
Gallop	4.0 ± 2.3	3.8 ± 2.5	4.3 ± 2.0			
Hop*	3.2 ± 2.4	2.8 ± 2.5	3.7 ± 2.3			
Leap*	2.7 ± 1.8	2.5 ± 1.8	3.1 ± 1.8			
Jump	4.8 ± 2.1	4.8 ± 2.3	5.0 ± 1.8			
Skip*	3.2 ± 2.3	2.6 ± 2.1	4.0 ± 2.4			
Slide	6.0 ± 2.1	5.8 ± 2.3	6.4 ± 2.0			
Strike				4.6 ± 2.0	4.7 ± 2.1	4.5 ± 1.9
Bounce				3.2 ± 1.6	3.3 ± 1.7	3.0 ± 1.6
Catch				4.7 ± 1.7	4.7 ± 1.7	4.8 ± 1.8
Kick*				4.2 ± 1.9	4.6 ± 1.8	3.8 ± 1.9
Throw				3.4 ± 1.9	3.6 ± 1.9	3.1 ± 1.9

*Denotes a significant difference between the girls and boys FMS scores (*P* < 0.05) (one-tailed)

**Table 4:** FMS competency score for locomotive, object control and total FMS. Data represent mean ± SD

Locomotive FMS			*Object control FMS			Total FMS		
All (*n* = 178)	Boys (*n* = 97)	Girls (*n* = 81)	All (*n* = 178)	Boys (*n* = 97)	Girls (*n* = 81)	All (*n* = 178)	Boys (*n* = 97)	Girls (*n* = 81)
31.5 ± 10.4	29.3 ± 10.5	34.2 ± 9.7	20.2 ± 6.1	20.8 ± 6.3	19.4 ± 5.9	51.7 ± 15.1	50.2 ± 15.5	3.5 ± 14.6

*Denotes a significant difference between the girls and boys FMS scores (*P* < 0.05) (one-tailed)

## Discussion

The current study extends understanding in this area, as it is the first study to examine differences in PA, BMI and WC, between preschool children, as a function of motor competency. This study discovered that none of the preschool children were considered ‘sufficiently active’ as none met the UK-recommended 180 min of PA per day [10]. This is supported by American and UK studies that found that preschool children did not accumulate sufficient PA for health benefits [39, 40, 45]. Conversely, Foweather et al. (2015) [15] reported that 86% of their sample met the recommended PA guidelines. This study similarly observed 3–5-year-old children from a deprived area, however, they were from a large urban city in the Northwest of England where geographical and environmental differences would have existed when compared to this current study. This could have influenced the differences in PA levels, yet further research into how and why is required. The study by Foweather et al. (2015) [15] did include a 6-week educational programme, which could have been a key determinant in the increased PA levels of the children they measured. Their assessment of PA was taken at two time points and did not cover all seasons, unlike the current study, which could have resulted in a difference in the reported PA levels between the two studies, whereas assessing across all the year would have provided a more representative assessment.

The key finding of this study is that there were no significant differences in PA level, BMI or WC, between preschool children classified as low, medium or high for locomotive, object control or total FMS. This study is novel in that it was first to use tertiles for FMS with preschool children. The results of this study add empirical evidence to suggestions made in Stodden et al.’s (2008) [51] conceptual model. In brief, the Stodden conceptual model [51] suggests that the association between motor competency and PA is weak in the developmental stage, but in older children, those with higher levels of motor competence and greater PA levels will perform better at both locomotor and object control skills. Therefore, the results of this current study support the findings of Stodden’s conceptual model [51], such that developing FMS competency is important in reducing sedentary behaviour and increasing PA for children’s future health. Children in the preschool years have not yet mastered their fundamental movement skills and there is considerable variability in movement patterns in children below the ages of 5 years. Proficiency in motor tasks during early childhood tends to be attained through free play and movement experiences but motor competence during this stage tends to be more rudimentary in nature [17]. In early childhood, direct experiences of PA promote motor skill development, and because children are afforded different PA experiences due to numerous constraints (e.g. environmental, economical) [37], a variation in motor proficiency will be observed. This makes the relationship between motor competency and PA less strong at this stage of childhood compared to those in middle and later childhood [51]. It could also be suggested that participation by only children from one deprived area resulted in the sample in the current study being too homogenous in terms of motor competence for a relationship between overall FMS and PA levels. That said, participants in this study had a total FMS score ranging from 6 to 82, with a mean score of 52 out of 90; suggesting that the participants were broadly representative of low, medium and high FMS competency.

Seefeldt (1980) [49] proposed a model of hierarchical order of motor skill development, which includes four levels: reflexes, fundamental motor skills, transitional motor skills and specific sport skills and dances. Progression through the levels occurs due to development in terms of growth, maturation and experience. Seefeldt (1980) [49] hypothesised that a ‘proficiency barrier’ exists between the fundamental and transitional levels of motor skill development in older children. Children who can achieve competency above the proficiency barrier will continue to engage in PA throughout their life, and those who do not exceed it are less likely to remain physically active. No proficiency barrier was found in this study with preschool children, unlike that demonstrated in older British children [12], thus, it provides an indication of a window to maximise input of good/optimal development of motor competence before the barrier sets in and we need remedial intervention.

This study was novel, as it assessed whether there is a relationship between weight status, PA levels and FMS of preschool children, using specific cut-points calibrated for use with UK preschool children, from a deprived area in the UK. As children age, those with intermediate to high levels of motor competence and greater PA levels will demonstrate higher performance scores [51]. The conceptual model [51] asserts that children with less developed FMS will enter a negative spiral of disengagement, develop lower levels of PA and have a greater chance of becoming obese during later childhood and adulthood. These children with lower FMS competency link to the proficiency barrier [49] in that they have not reached a certain level of maturity/competency, therefore they will not continue to engage in PA. In this study, there was no significant relationship between FMS competency and the percentage of time spent in sedentary, light, moderate or vigorous PA or with weight status. Studies examining preschool children similarly report no association or a weak relationship [13, 42] with variation in FMS proficiency, only explaining 3% of the variation in PA levels in preschool children [42]. These findings are likely to be because PA and FMS may not be fully established and consequently not strongly linked at the ages of 3–4 years. Minimal studies have investigated the relationship between FMS and PA in preschool children and further studies are needed to support this assertion.

The current study demonstrated that there was no significant difference in BMI or WC between preschool children split into tertiles of FMS competency, unlike research with older children. Older overweight children, due to their increased mass, have greater difficulty when performing motor skills, especially locomotor skills [21]. Therefore, higher mass results in lower motor competence in older children [51]. The Stodden et al. (2008) [51] model proposes that as a child enters middle childhood, their perceived motor skills competency starts to change as they compare themselves to their peers. Therefore, a less competent child will possess lower perceived competency and this reflects their actual motor competency, such that these children will then start to opt out of PA and enter a negative spiral of disengagement in PA, whereby motor skill competence, physical fitness and PA are all low, leading to increased weight and obesity. However, this current study found no difference in BMI/WC between FMS tertiles in pre-school children. This could be as a result of FMS focusing on the assessment of the movement skills performance, not product, over a short time [29]. Therefore, these skills (catching, throwing, running) are independent of physical fitness (cardiovascular and muscle endurance) and physical features, such as weight and height. Additionally, assessment tools such as the TGMD-2 require the children to possess a cognitive understanding regarding the FMS. This can be highlighted through catching, as children who show well-developed catching skills without awareness of the preparation phase will receive a low score [29]. This is significant for preschool children as these findings would suggest that differences in FMS mastery are more related to their cognitive abilities and physiological responses than their motor abilities; this is an area for future examination.

The current data shows that a substantial proportion of each day is spent in sedentary behaviour in British preschool children. This may explain the lack of any significant differences in PA levels between tertiles of FMS competency, as the majority of the participants in this study participated in sedentary behaviour (545 ± 85 min and 94.2 ± 2.8%) on both the week and weekend days (overall PA). If there was more variability across the sample in how much time was spent in MVPA, possibly via the use of a different sample of children or through a specific intervention, then there is the possibility that significant differences in PA levels and weight status between tertiles of FMS competency might occur; this, therefore, requires further investigation.

The girls in the present study were predominantly better at the locomotor skills, which are associated with more coordinated movements [8] and the boys were predominantly better at the object control skills, which is consistent with existing literature [15]. Gender differences during the preschool years cannot solely be explained by biological factors, as socialisation can explain variations between genders in FMS competency [23], with girls’ overall PA levels affected by each other and the environment they are in [44]. This highlights the necessity for a variety of more structured activities, to be promoted for preschool girls throughout their day. It was reported that there was no correlation between the FMS subsets of locomotor and object control FMS and MVPA during weekdays and weekend days. Equally, there was no correlation between these FMS subsets and BMI or WC; supporting Stodden et al.’s (2008) [51] view that preschool children are still in the developmental stage. Therefore, allowing preschool children further practice and instruction to reach mature patterns of movement ready for primary school is essential. Future research could identify if such an intervention results in greater PA levels and improved weight status.

### Practical considerations

Using wrist-worn accelerometers can be logistically and practically challenging with preschool children [8]. In the current study, although a cut-off of 6 h per day was employed, the participants, in general, far exceeded this value with mean wear times being over 600 min/day for both weekdays and weekends. This may be considered a comparatively low wear time, however, achieving a greater amount of days and higher wear time in preschool children is challenging. The preschool children were drawn from a deprived area of the UK. This may have resulted in differences in PA levels and FMS competency rate in the study, compared to an area of higher SES, therefore future research comparing both high and low SES groups would be welcome. That said, the focus on low SES children was important as these children face greater barriers to becoming physically active and when older they face higher rates of obesity and associated comorbidities [35]. It is important to emphasise that the participant’s preschools were all in a deprived area, however, the samples were probably not homogenous in terms of their home social and physical environment, as not all children were necessarily from a low SES household. This study did not assess the interaction between familiar environments, PA levels of parents, ponderal status of parents or genetics of the children, but these would be key areas to focus on in future research to identify if they are contributing factors to a preschool child’s FMS competency. Also, none of the preschool children met the UK-recommended PA guidelines, but the majority were of a healthy weight. This in itself is a key consideration and may indicate a window of opportunity for PA intervention before overweight/obesity has set in. Finally, a single accelerometer may not capture all PA at these ages, as preschool children may play a lot on the ground without displacement and when they walk, they may not move their upper limbs with a regular pattern, therefore the wrist-worn GENEActiv accelerometer may not capture all movements, thereby potentially underestimating total physical activity. However, use of a single accelerometer has previously been calibrated for use in this population [47].

## Conclusions

Preschool children from low SES/deprived areas are extremely inactive in their behaviours, as none of the preschool children in this sample achieved the UK-recommended guidelines of PA for health. FMS competency did not appear to influence the level of PA or weight status in this sample of UK preschool children from a low SES area. This is potentially because PA and FMS may not be fully established and consequently not strongly linked at the ages of 3–4 years. Therefore, the preschool years could be influential in providing a window to maximise input of good/optimal development of motor competence before the proficiency barrier sets in and we need remedial intervention. Equally, the preschool children in this study had low PA levels, therefore, further research is required to identify if a relationship with FMS may only be evident when children are more active.

## Abbreviations

ANOVA: Analysis of variance
BMI: Body mass index
FMS: Fundamental movement skills
LPA: Light physical activity
MVPA: Moderate to vigorous physical activity
PA: Physical activity
SES: Socio-economic status
SPSS: Statistical Package for Social Sciences
TGMD-2: Test of Gross Motor Development-2
WC: Waist circumference
